# Report of a Case of Video-Assisted Thoracoscopic Resection of Bronchogenic Cyst Developed in the Aorto-Pulmonary Window

**DOI:** 10.1155/DTE.3.111

**Published:** 1996

**Authors:** T. De Giacomo, F. Venuta, E. A. Rendina, E. Guarino, I. Flaishman, C. Ricci

**Affiliations:** University of Rome “La Sapienza” Department of Thoracic Surgery II Surgical Clinic Policlinico Umberto I, V. le Policlinico Rome 00164 Italy

## Abstract

We report the case of a 28-years-old male with a bronchogenic cyst developed in the aorto-pulmonary window. Left video-assisted thoracoscopy was performed and the cyst was removed intact and completely. Operative time was 48 minutes. The postoperative course was uneventful and the patient was discharged on the third postoperative day. We believe that an uncomplicated mediastinal bronchogenic cyst can be successfully approached by video-assisted thoracoscopy. In the case of an intraparenchymal or complicated cyst, thoracoscopic resection can be technically difficult and hazardous, and open approach is preferable.


*Diagnostic and Therapeutic Endoscopy*, 1996, Vol. 3, pp. 111–113
Reprints available directly from the publisher
Photocopying permitted by license only

© 1996 OPA (Overseas Publishers Association)
Amsterdam B.V. Published in The Netherlands
by Harwood Academic Publishers
Printed in Malaysia

### Report of a Case of Video-Assisted Thoracoscopic Resection of Bronchogenic Cyst Developed in the Aorto-Pulmonary Window

T. DE GIACOMO, F. VENUTA, E. A. RENDINA, E. GUARINO,
I. FLAISHMAN, and C. RICCI

*University of Rome "La Sapienza", Department of Thoracic Surgery, II Surgical Clinic, Policlinico Umberto I, V. le Policlinico, 00164, Rome, Italy*

*(Received 21 December 1995; In final form 8 July 1996)*

**We report the case of a 28-years-old male with a bronchogenic cyst developed in the aorto-pulmonary window. Left video-assisted thoracoscopy was performed and the cyst was removed intact and completely. Operative time was 48 minutes. The postoperative course was uneventful and the patient was discharged on the third postoperative day. We believe that an uncomplicated mediastinal bronchogenic cyst can be successfully approached by video-assisted thoracoscopy. In the case of an intraparenchymal or complicated cyst, thoracoscopic resection can be technically difficult and hazardous, and open approach is preferable.**

*Keywords:* Thoracoscopy, bronchogenic cyst

### INTRODUCTION

Bronchogenic cysts, account for 10 to 15% of all mediastinal tumors and about 60% of mediastinal cysts [1,2]. These lesions are commonly located posterior to the carina, but can be found within lung parenchyma in connection with bronchi, in the pulmonary hilum or closely associated with the esophagus. Bronchogenic cysts are usually single but may be multilocular or multiple. The wall of the cyst is composed of fibroelastic tissue and cartilage; the lining generally consists of pseudostratified epithelium. Bronchogenic cysts are seldom seen in the adult and most are initially asymptomatic. Surgical resection is indicated because the majority of the cysts will ultimately become symptomatic or complicated. Video-assisted thoracoscopy offers the possibility of treating this lesion postoperative in selected cases, with low invasivity, low pain and shorter recovery time. We report the case of thoracoscopic resection of uncomplicated cysts developed in the aorto-pulmonary window.

*Corresponding author. Fax: 0039/6/49970735.

### MATERIAL AND METHODS

A 28-years-old male, was referred to our department, complaining of chest pain and mild dyspnea. Physical examination was unremarkable. Chest X-ray showed an enlargement of the left mediastinal shadow and computed thomography of the chest confirmed the presence of a 3-cm lesion, with fluid density, developed in the aorto-pulmonary window closely associated with 2 enlarged lymph nodes (Fig. 1). Fiberoptic bronchoscopy was negative and complete blood count, lung function tests and blood gas analysis were normal. Under general anesthesia with double lumen intubation, the patient was placed in a right lateral decubitus position. The operative field was prepared and draped as for left thoracotomy. A 11-mm trocar was inserted through the VII intercostal space along the midaxillary line. The thoracoscope was introduced and the left chest cavity was explored. No abnormalities of pleural surfaces and of the lung were discovered. Two more trocars were positioned respectively through the IV and VI intercostal spaces along the anterior axillary line. The lung was retracted posteriorly with the help of the back rotation of the operating table, and the aorto-pulmonary window was exposed and the lesion identified. It appeared soft and well circumscribed. Sharp and blunt dissection was employed to detach the cyst from the surrounding structures and to separate the lesion from the underlying pulmonary artery. Special attention was paid to avoid the rupture of the cyst during the dissection and to preserve the laryngeal nerve. Endoscopic clips were used for the hemostasis of small vessels. After complete excision, the cyst was placed in a surgical glove introduced into the chest through one of the trocar sites and the intact gloved specimen was removed through the axillary trocar incision, slightly enlarged. Two mediastinal nodes (station 5) were resected. A benign cyst lined with pseudostratified epithelium and reactive inflammatory nodes were demonstrated by frozen section. Chest tube drainage was inserted through the lower trocar site and the other thoracic incisions were sutured. Total operative time was 48 minutes. The patient's postoperative course was uneventful, and the chest tube was removed in the II postoperative day. Prophylactic antibiotic therapy was administred until the removal of the chest tube. Postoperative pain

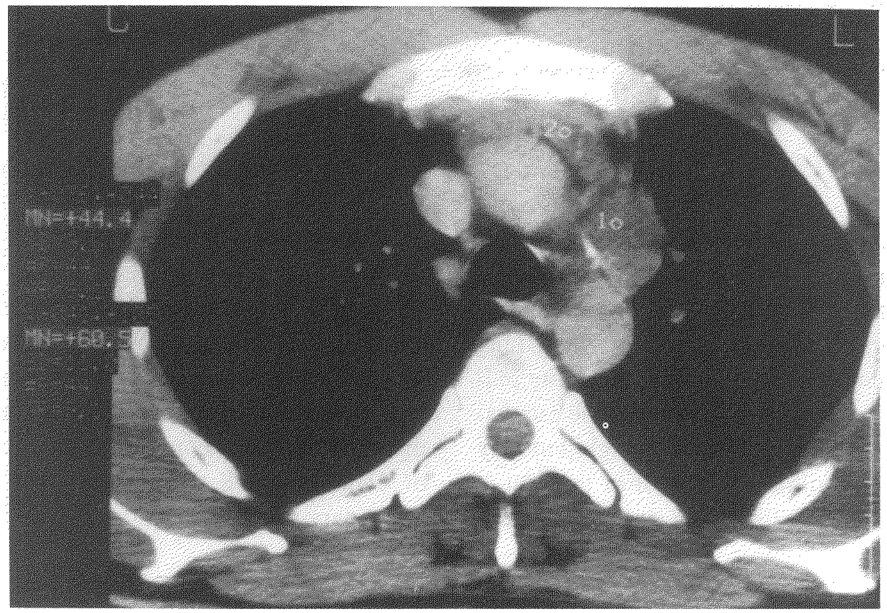

FIGURE 1 Chest CT-Scan shows a 3 cm lesion with a fluid density in the aorto-pulmonary window, associated with 2 enlarged nodes.

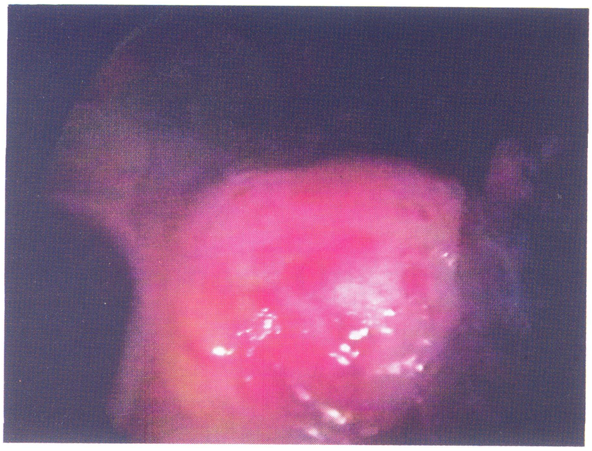

FIGURE 2 Thoracoscopic aspect of the bronchogenic cyst.

required only nonsteroidal analgesics. Culture for bacteria and fungus were negative and the patient was discharged on the third postoperative day. After a follow-up of 18 months, there is no evidence of cyst recurrence.

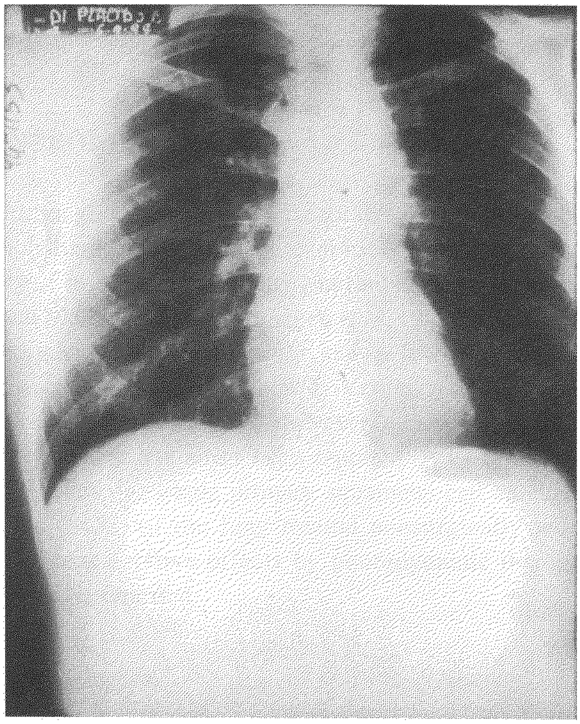

FIGURE 3 Postoperative chest X-ray.

### DISCUSSION

Video-assisted thoracoscopy (VAT) offers the possibility for diagnosis and treatment of many different diseases of the chest. Thoracoscopic resection of bronchogenic cysts have already been reported [2,3,4], but as far as we know, this is the first case of described cyst developed in the aorto-pulmonary window resected thoracoscopically. We were able to resect the lesion completely, removing it intact avoiding the risks of pleural contamination and recurrence. These conveniences can be due to cyst disruption during the dissection, and to an uncomplete resection. Thoracoscopy is certainly less invasive than thoracotomy, with low postoperative pain and shorter recovery time. Neverthless, this approach should be employed in selected cases for uncomplicated mediastinal cysts. In fact, the majority of bronchogenic cysts developed within the lung are in connection with the airway and often they are infected and symptomatic. Mediastinal cysts can also become infected or can present intrinsic complications such as inflammatory changes, ulceration and hemorrage. In these cases, thoracoscopic resection can be hazardous because of dense pericystic adhesions with the surrounding structures and for the risk of pleural contamination.

In conclusion, we believe that all bronchogenic cysts should be resected because the majority will become symptomatic or complicated. Thoracoscopic approach is effective and safe for resecting uncomplicated mediastinal bronchogenic cysts. In our opinion, open approach should be employed for complicated or intra-parenchymal lesions.

### *References*

[1] Razemon, P. and Ribet, M. (1990). Chirurgie du Mediastin, *Masson Ed.*, Paris.

[2] St. Giorge, R., Desaluriers, J., Duranceau, A., Vaillancourt, R., Deshamps, C., Beauschamp, G., Pagè, A. and Brisson, J. (1991). Clinical spectrum of bronchogenic cysts of the mediastinum and lung in the adult, *Ann. Thorac. Surg.*, **52**, 6–13.

[3] Lewis, R. J., Caccavale, R. J. and Sisler, G. E. (1992). Imaged thoracoscopic surgery: a new technique for resection of mediastinal cyst, *Ann. Thorac. Surg.*, **53**, 318–20.

[4] Naunheim, K. S. and Andrus, C. H. (1993). Thoracoscopic dreinage and resection of giant mediastinal cyst, *Ann. Thoracic. Surg.*, **55**, 156–158.

